# Characterization and Biological Activities of Melanin from the Medicinal Fungi *Ophiocordyceps sinensis*

**DOI:** 10.3390/ijms241210282

**Published:** 2023-06-17

**Authors:** Chaoqun Tong, Jian Luo, Chaolu Xie, Junhong Wei, Guoqing Pan, Zeyang Zhou, Chunfeng Li

**Affiliations:** 1 State Key Laboratory of Resource Insects, Southwest University, Chongqing 400715, China; tcqphd@email.swu.edu.cn (C.T.); jianluo0214@163.com (J.L.); 13028325332@163.com (C.X.); weijunhong@swu.edu.cn (J.W.); gqpan@swu.edu.cn (G.P.); 2 Chongqing Key Laboratory of Microsporidia Infection and Prevention, Southwest University, Chongqing 400715, China; 3 Key Laboratory of Tropical Translational Medicine of Ministry of Education, Hainan Medical University, Haikou 571199, China; 4 NHC Key Laboratory of Tropical Disease Control, Hainan Medical University, Haikou 571199, China; 5 College of Life Sciences, Chongqing Normal University, Chongqing 401331, China

**Keywords:** *Ophiocordyceps sinensis*, melanin, structural characterization, antioxidant activity, metal ions adsorption

## Abstract

Melanin is a complex natural pigment that is widely present in fungi. The mushroom *Ophiocordyceps sinensis* has a variety of pharmacological effects. The active substances of *O. sinensis* have been extensively studied, but few studies have focused on the *O. sinensis* melanin. In this study, the production of melanin was increased by adding light or oxidative stress, namely, reactive oxygen species (ROS) or reactive nitrogen species (RNS), during liquid fermentation. Subsequently, the structure of the purified melanin was characterized using elemental analysis, ultraviolet-visible absorption spectrum, Fourier transform infrared (FTIR), electron paramagnetic resonance (EPR), and pyrolysis gas chromatography and mass spectrometry (Py-GCMS). Studies have shown that *O. sinensis* melanin is composed of C (50.59), H (6.18), O (33.90), N (8.19), and S (1.20), with maximum absorbance at 237 nm and typical melanin structures such as benzene, indole, and pyrrole. Additionally, the various biological activities of *O. sinensis* melanin have been discovered; it can chelate heavy metals and shows a strong ultraviolet-blocking ability. Moreover, *O. sinensis* melanin can reduce the levels of intracellular reactive oxygen species and counteract the oxidative damage of H_2_O_2_ to cells. These results can help us to develop applications of *O. sinensis* melanin in radiation resistance, heavy metal pollution remediation, and antioxidant use.

## 1. Introduction

Melanin is a dark-colored polymer of phenols and indoles that is widely distributed among animals, plants, and microorganisms. Melanin can be categorized into eumelanin, phaeomelanin, and allomelanin based on its color and elemental composition [1]. Eumelanin is black or dark brown, contains nitrogen elements but not sulfur elements, and mainly exists in microorganisms and the eyes and fur of animals; phaeomelanin is red, brown, or yellow, contains nitrogen and sulfur elements, and mainly exists in animal hair; allomelanin is black or brown, sometimes contains a small amount of nitrogen, and generally exists only in plants and fungi [2,3,4]. The precursors of melanin include DOPA, DHN (1,8-dihydroxynaphthalene), 5,6-dihydroxyindole, catechol, and HGA (homogentisic acid), it has extremely complex and diverse chemical structures [5].

Melanin contains multiple functional groups and complex structures, which help to resist extreme temperature, ultraviolet (UV) radiation, heavy metal ions, and other environmental stresses, and is widely used in the fields of food, cosmetics, optoelectronic biomaterials, and ecological restoration [6,7]. Various studies have reported many biological activities of melanin. Melanin isolated from *Lachnum* YM226 lowered blood lipid levels and showed antitumor activity in a mouse model [8]. The melanin synthesized from *Inonotus hispidus* has a strong antioxidant capacity, which can significantly reduce reactive oxygen species (ROS) in LO2 cells and thus protect the liver from oxidative damage [9]. Melanin also has antibacterial, anti-radiation, DNA protection, and other physiological activities [10,11,12].

The requirement for melanin has increased rapidly due to its extensive biological activity [1,13]. Through microbial fermentation, melanin can be extracted quickly, cheaply, and on a large scale to meet market demands [5,14]. Fungi, especially edible and medicinal fungi (mushrooms), are excellent sources of natural melanin because they are safe to consume [15]. *Ophiocordyceps sinensis*, a fungus belonging to Ascomycota, Sordariomycetes, Hypocreales, Ophiocordycipitaceae, and *Ophiocordyceps*, is the anamorph of Chinese cordyceps and a precious Chinese traditional medicine. *O. sinensis* has many pharmacological effects such as antioxidative, anti-inflammatory, hypoglycemic, and immunomodulatory activities [16,17,18,19]. Previous studies have found that *O. sinensis* can synthesize melanin during liquid fermentation; it shows good antioxidant activity against 2,2-diphenyl-1-picryl-hydrazyl (DPPH) and Fe^2+^ chelating activity [20]. However, the structure of *O. sinensis* melanin has not been analyzed in detail, and its biological activity needs to be further explored.

In this study, we showed that the production of *O. sinensis* melanin was significantly increased by inducing NO stress with sodium nitroferricyanide dihydrate (SNP). The melanin structure was characterized using full wavelength scanning absorption spectroscopy, Fourier transform infrared spectroscopy (FTIR), elemental analysis, and pyrolysis gas chromatography and mass spectrometry (Py–GCMS). The data on the structure of melanin can help us to better understand its physiological activity. The structure of melanin varies among different species, resulting in different physiological functions. This study found that the structure of *O. sinensis* melanin is complex; in addition, we preliminarily demonstrated that *O. sinensis* melanin has multiple biological and physiological activities, such as blocking UV radiation, chelating metal ions, and clearing intracellular ROS to inhibit cell apoptosis.

## 2. Results

### 2.1. Induction Conditions of Melanin

**Figure 1:**
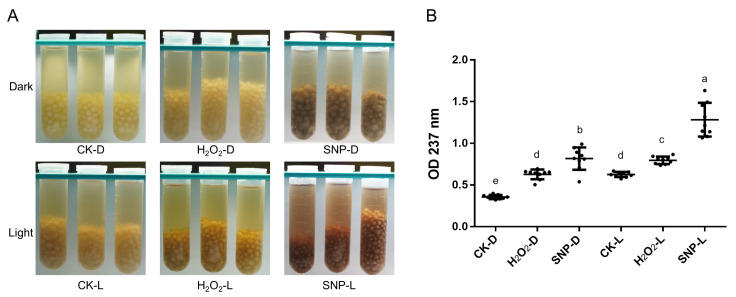
Effects of light and oxidative stress on melanin synthesis. (**A**) Color phenotypes of fermented liquid and mycelial pellets under light-induced and oxidative stress (100 μmol/L H_2_O_2_ or SNP). (**B**) Quantitative analysis of melanin yield under different induction conditions. Each point represents one experimental datum, and each group consists of three biological replicates and three technical replicates. D and L represent dark and light conditions, respectively. Different lowercase letters indicate significant differences between groups (*p* < 0.05).

*O. sinensis* proliferated in the form of hyphal aggregates in OS1 medium. The hyphal aggregates were white in the dark, and the color of the culture medium did not appreciably change. The color of the hyphal aggregates and the culture medium considerably darkened under light-induced circumstances (Figure 1A). Similarly, the addition of H_2_O_2_ and SNP, which induce ROS and reactive nitrogen species (RNS) oxidative stress, could also stimulate the production of melanin. After the fermentation products under different conditions were extracted for melanin, the absorbance was measured at 237 nm. The results showed that the applying of RNS stress significantly promoted melanin yield (Figure 1B).

### 2.2. Elemental Analysis

Despite the structure complexity of fungal melanin, it can be generally categorized depending on the composition and content of elements. The elemental analysis of *O. sinensis* and other fungi melanin are shown in Table 1. The percentages of C, H, O, N, and S in *O. sinensis* melanin were 50.59%, 6.18%, 33.90%, 8.19%, and 1.20%, respectively.

Melanin extracted from *O. sinensis*, *Ganoderma lucidum*, and *Boletus griseus* were similar and contained a small amount of S; they are likely to be eumelanin. But *O. sinensis* melanin contained lower C/N, C/H, and C/O, indicating that it may contain more heterocycles or fatty groups.

### 2.3. Spectral Characterization

**Figure 2:**
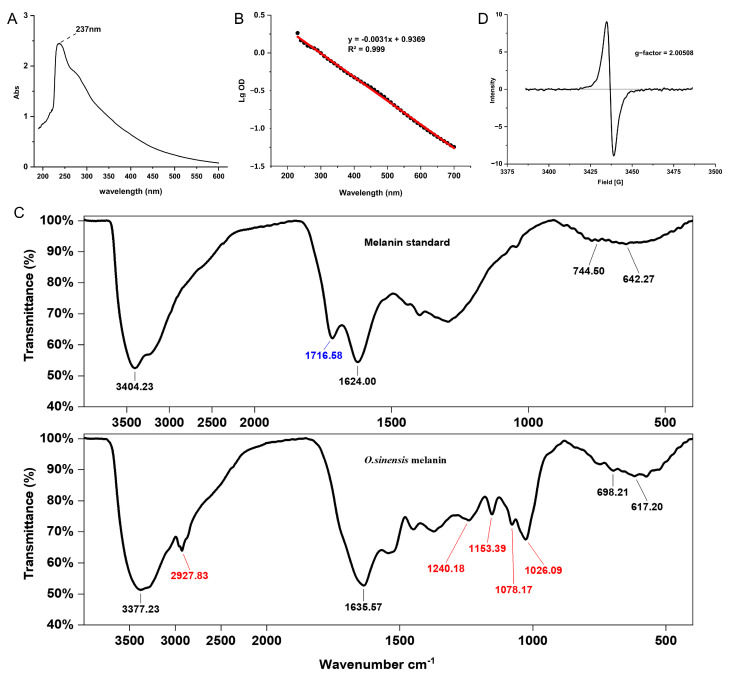
The spectroscopy analysis of *O. sinensis* melanin. (**A**) UV-visible absorption spectra. (**B**) The lg value of absorbance at different wavelengths. The black dots represent the measured data points, and the red line represents the linear fitting result. (**C**) Fourier transform infrared spectra. (**D**) Electron paramagnetic resonance spectra.

The UV-visible spectrum of *O. sinensis* melanin exhibited strong optical absorbance in the UV region, and the maximum absorption peak was observed at 237 nm (Figure 2A). In addition, the log of the optical density of *O. sinensis* melanin against its wavelengths produced a linear curve with a negative slope of −0.0031, which is consistent with the absorption characteristics of melanin (Figure 2B), indicating that the purity of the extracted melanin was high.

The FTIR spectrum of *O. sinensis* melanin and qualitative standard displayed several typical spectral bands (Figure 2C). The strong and broad complex bands at 3400 cm^−1^ corresponded to the -NH group connected to the -OH group of the indole ring, which is the characteristic group of melanin. Compared with the qualitative standard, *O. sinensis* melanin had a unique absorption peak at 2930–2830 cm^−1^, combined with the weak absorptive peak at 1240 cm^−1^, indicating an asymmetric vibration of -CH_2_. The peak at 1720–1715 cm^−1^ corresponded to ketones, indicating that the standard substance contains C=O, while *O. sinensis* melanin does not contain this group. The peak at 1650–1620 cm^−1^ corresponded to the asymmetric deformation vibration of C=O and N-H in amide, indicating the presence of a substituted carboxyl. The weak peaks at 1150 cm^−1^ corresponded to the amino group C-N. The peak at 1078 cm^−1^ corresponded to the stretching vibration of C-O in phenol or carboxyl. Because the *O. sinensis* melanin contains the element S, it may also be a sulfonic acid group -SO_3_H. The peak at 1026 cm^−1^ corresponded to the skeletal vibration of C-CH_3_. The peaks at 800–600 cm^−1^ were weak, indicating that the aromatic ring was replaced to form a conjugated system with low aromatic hydrogen. The above characteristics indicate that the infrared spectrum of *O. sinensis* melanin obtained through extraction and purification conforms to the structural characteristics of traditional melanin. 

The EPR spectrum of *O. sinensis* melanin showed a strong single slightly asymmetric line, indicating the presence of stable organic free radicals, and the g-factor was 2.00508 (Figure 2D).

### 2.4. Py-GCMS Analysis

Py-GCMS can provide monomer information about complex compounds from their pyrolysis products; therefore, it is perfectly suited for the structural characterization of melanin [25]. In this study, Py-GCMS was used to further characterize the structure of *O. sinensis* melanin, and a total of 24 compounds were identified (Table 2). Benzene, indole, and pyrrole were detected in *O. sinensis* melanin (components 4 and 10), which are the characteristic products of the thermal decomposition of eumelanin [26]. Moreover, furan was detected in *O. sinensis* melanin (component 19), which is similar to *B. griseus* melanin [25]. In addition, the high abundance of alkanes in the thermal cracking products of *O. sinensis* melanin indicates there are more saturated bonds, which is consistent with the lower C/N and C/H in the elemental analysis (Table 1).

### 2.5. Ability to Chelate Metal Ions

**Figure 3:**
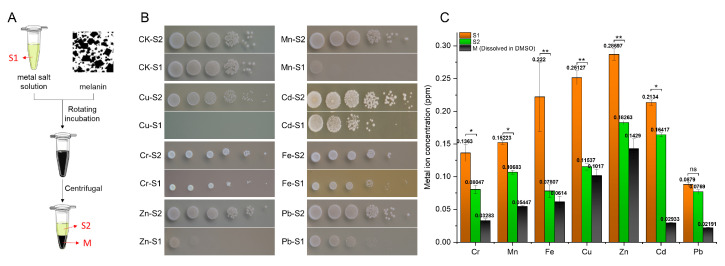
The metal-ion-chelating ability of *O. sinensis* melanin. (**A**) Schematic diagram of metal ion chelation experiment. (**B**) Inhibition effect of the metal solution on *S. cerevisiae* growth. (**C**) Concentration of metal ions in each component during the incubation experiment. Values represent the mean ± SD of three experiments; ANOVA was used to compare the concentration of metal ions in the solution of melanin before (S1) and after (S2) incubation. (* *p* < 0.05; ** *p* < 0.01; ns, no significant difference).

The ability to chelate metal ions of *O. sinensis* melanin was explored through coincubation experiments (Figure 3A). It was found that the inhibiting effect of metal solutions on *S. cerevisiae* cell growth was greatly diminished following *O. sinensis* melanin incubation (Figure 3B). Furthermore, the metal ion concentration in the preincubation and postincubation supernatant metal solutions and precipitate melanin solutions (dissolved in DMSO) was detected; the results showed that melanin can transfer metal ions from supernatant metal solutions to precipitate melanin solutions (dissolved in DMSO) through chelation (Figure 3C).

### 2.6. UV-Blocking Activity

**Figure 4:**
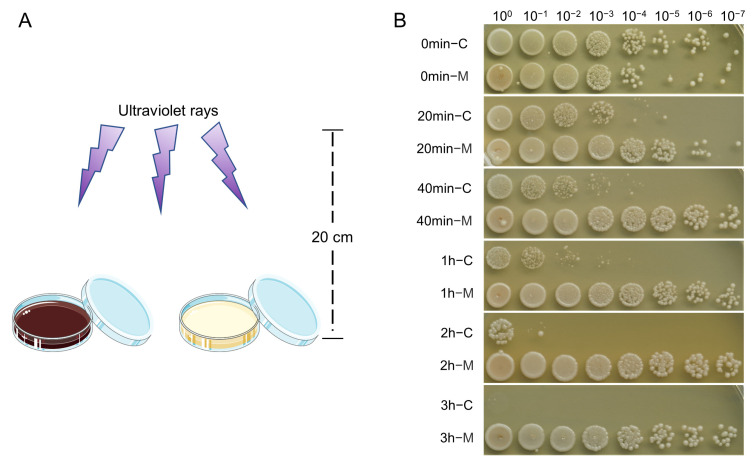
The UV-blocking ability of *O. sinensis* melanin. (**A**) Schematic diagram of UV-blocking experiment. (**B**) Vitality of *S. cerevisiae* cells at different times of UV radiation.

*S. cerevisiae* was mixed with melanin solution (0.1% DMSO as a control), and irradiated under UV light at different times (Figure 4A). The survival rate of yeast cells was used to evaluate the resistance to UV radiation. The result showed that, after 20 min of UV radiation, the survival rate of the control group decreased significantly, and all the yeast cells died after 3 h exposure to UV light. In contrast, the survival of yeast cells mixed with *O. sinensis* melanin was essentially unaffected; its survival rate still kept comparable to the unirradiated group even after 3 h of UV irradiation. The results indicated that melanin can block the damage of UV radiation and free radicals caused by UV-generated ozone (Figure 4B).

### 2.7. Antioxidant Activity

**Figure 5:**
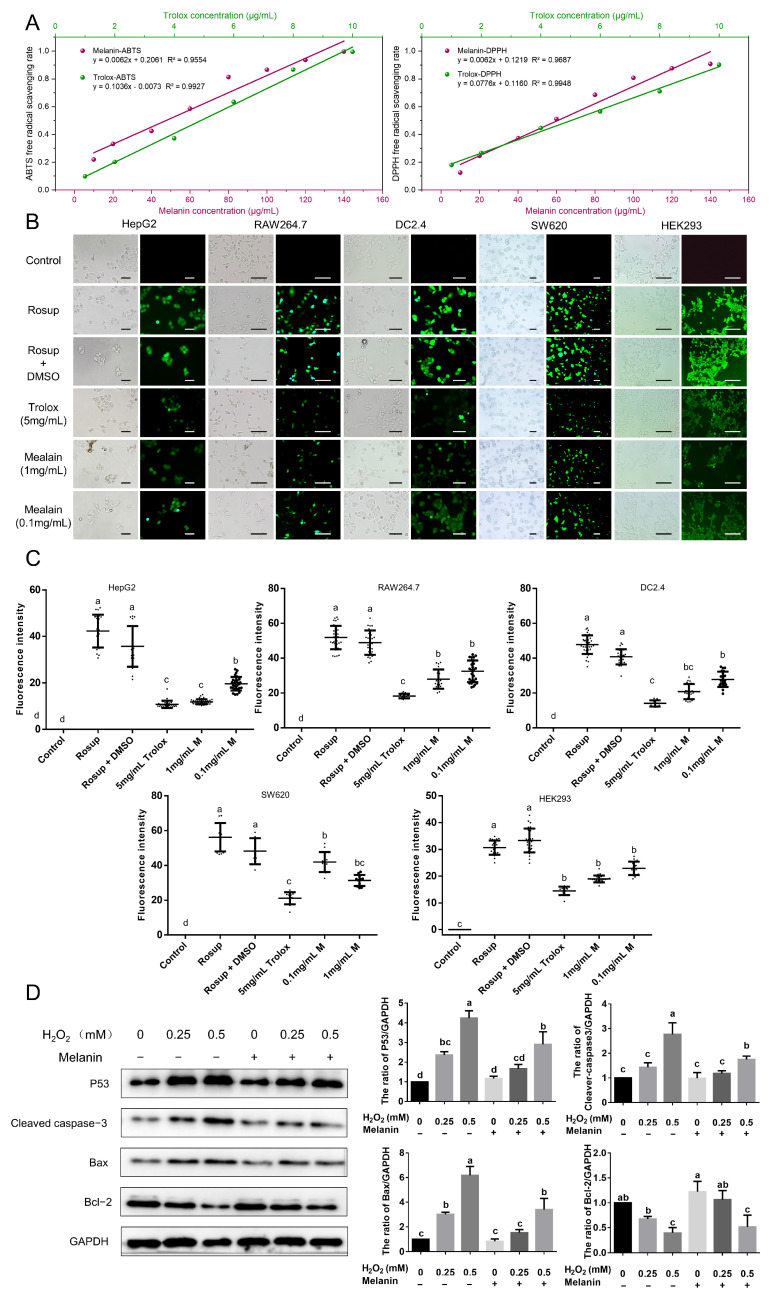
Antioxidant activity of *O. sinensis* melanin. (**A**) ABTS and DPPH radical scavenging activities. (**B**) The scavenging effect of *O. sinensis* melanin on intracellular ROS; green fluorescence indicates DCFH-DA labeled ROS. Bar = 20 μm. (**C**) Quantification and statistical analysis of the scavenging effect of *O. sinensis* melanin on intracellular ROS. (**D**) Preincubation with *O. sinensis* melanin reduces the apoptosis induced by H_2_O_2_ in HEK293 cells. Different lowercase letters indicate significant differences between groups (*p* < 0.05).

The free-radical-scavenging assay of *O. sinensis* melanin is shown in Figure 5A. Both DPPH and ABTS free radicals could be scavenged by *O. sinensis* melanin, and the activity was dose-dependent. In this assay, the radical-scavenging activity of *O. sinensis* melanin was lower than that of Trolox.

Moreover, the activity of *O. sinensis* melanin in removing intracellular ROS was examined using five cell lines, and the level of intracellular ROS was shown by the DCF fluorescence intensity. The use of an active oxygen inducer (Rosup) dramatically enhanced intracellular ROS, and when the cells were preincubated with *O. sinensis* melanin, the levels of intracellular ROS were greatly inhibited, and this action was dose-dependent (Figure 5B). The quantitative analysis of DCF fluorescence intensity revealed that *O. sinensis* melanin has a stronger ability to neutralize intracellular ROS than Trolox, indicating a strong antioxidant capacity (Figure 5C).

It is well established that treatment of HEK293 cells with H_2_O_2_ causes an increase in ROS, which lead to apoptosis. To analyze the protective effect of *O. sinensis* melanin on H_2_O_2_-mediated cell damage, Western blotting was used to detect the expression of the apoptotic-related proteins. The results showed that the expression of the apoptosis-inhibitory protein Bcl-2 decreased after H_2_O_2_ treatment, while pretreatment with *O. sinensis* melanin could restore the expression level of Bcl-2 (Figure 5D). In contrast, the apoptosis-related protein P53, the apoptosis-marker-cleaved caspase-3, and the proapoptotic protein Bax were down-regulated after being pretreated with *O. sinensis* melanin. These results indicate that *O. sinensis* melanin can exert antiapoptotic effects by neutralizing ROS.

## 3. Discussion

*O. sinensis* is an important edible medicinal fungus; its active substances such as polysaccharides and peptides have been extensively studied [17,19], but few studies have focused on the medicinal value of *O. sinensis* melanin. Melanin is a crucial part of the fungal cell structure and has a variety of physiological and biochemical functions [14]. Numerous fungi, including *I. hispidus*, *B. griseus,* and *Auricularia auricula*, have been used to produce melanin, and their structures and functions have been studied [9,25,27]. Reports of preliminary studies on the structure and function of *O. sinensis* melanin indicate that it shows antioxidant activities [20]. In this study, we initially explored the induction conditions of *O. sinensis* melanin, and the structural characteristics of *O. sinensis* melanin were detected using elemental analysis, UV-visible absorption spectroscopy, FTIR spectroscopy, and Py-GCMS. In addition, we studied the biological activities of *O. sinensis* melanin such as chelating metal ions, UV blocking, and antioxidant properties.

The average yield of melanin varies greatly among different fungal species; for example, it is 0.54 mg/g in *Phoma* sp. RDSE17 and 0.938 g/L in *Hortaea werneckii* AS1 [22,28]. Generally, the production of fungi melanin is not very high; therefore, the culture medium and conditions need to be optimized to boost melanin production [29]. *Aspergillus niger* intensely accumulated melanin under light, suggesting that light can be used as an inducer to increase melanin production [30]. It is widely believed that fungi can perceive and respond to light signals. On the one hand, fungi can respond to light signals through photoreceptor proteins, thereby initiating the transcription of downstream genes. On the other hand, light stress can also directly induce intracellular ROS, leading to changes in the physiological balance of cells and thereby triggering a series of antagonistic reactions [31]. Similarly, in this study, the yield of *O. sinensis* melanin could be increased under light or oxidative stress (ROS and RNS).

Analysis of the chemical structure of melanin helps to understand its biological activity. It has been reported that fungi can synthesize allomelanin through the DHN pathway and synthesize pheomelanin (contains 9–12% S) and eumelanin (contains 0–1% S) through the DOPA pathway [32]. *O. sinensis* melanin contains 1.2% S, and the C/N ratio is 7.21, which conforms more to eumelanin. In the UV-visible absorption spectrum, *O. sinensis* melanin has maximum absorption values at 237 nm, and a plot of the log of absorption against wavelength is a straight line. The FTIR spectroscopy showed the characteristic absorption peaks of *O. sinensis* melanin. Combined with the Py-GCMS results, we demonstrated that *O. sinensis* melanin contains benzene, indole, pyrrole, and furan, which are the most significant thermal degradation products of eumelanin [25]. Compared with dopa melanin standards, *O. sinensis* melanin contains more saturated bonds, which is consistent with the lower C/N and C/H ratios in the elemental analysis. Through elemental analysis, the results showed that *O. sinensis* melanin has a high N element content and a very low C/N ratio when compared with other fungal melanin. In conjunction with Py-GCMS, we found that there are a lot of -NH_2_ and -NH in *O. sinensis* melanin. As -NH_2_ is an active and easily oxidized group, these results further indicated the potent antioxidant properties of *O. sinensis* melanin.

Melanin contains numerous functional groups, such as -OH, -COOH, and -NH, which make it an ideal choice for heavy metal remediation [33]. Therefore, melanin can fix metal ions through chelation to reduce the harm caused by heavy metals to organisms. *Azotobacter chrooccum* can synthesize dark brown melanin, which can chelate Cd^2+^, Cr^2+^, and Ni^2+^. Melanin not only makes *A. chrooccum* highly metal tolerant but also protects plants in heavy-metal-polluted environments [34]. Owing to the diversity of its structure, different melanin has different characteristics for adsorbing metal ions. *Pseudomonas stutzeri* melanin has a higher adsorption capacity for Cu^2+^ and Pb^2+^ than Hg^2+^, while *Ommastrephes bartrami* melanin has a higher adsorption capacity for Pb^2+^ and Cd^2+^ [35,36]. The adsorption ability of *O. sinensis* melanin for seven heavy metals was investigated in this study, and the results indicated that *O. sinensis* melanin has the best adsorption capacity for Pb^2+^, Cd^2+^, and Mn^2+^, but a poor adsorption capacity for Fe^2+^ and Cu^2+^.

In general, melanin exists in the form of insoluble granular particles and is localized on the fungal cell wall. Numerous studies of melanin using microscopy demonstrated that melanin is cross-linked with polysaccharide components to form the components of spore walls. This is also the reason why high-temperature and strong alkaline treatments are necessarily used to separate melanin from the stable spore wall structure [37,38]. Studies have shown that melanin is stored as granules within the reticular scaffold of chitin aggregation; therefore, the mutation of chitin synthase will lead to the disruption of the structure of the fungal cell wall and the release of melanin into the culture medium [39,40]. When *O. sinensis* melanin is induced, the color of the culture medium will change from faint yellow to black; this indicates that some melanin is secreted into the culture medium. The secretory melanin is likely to have other functions due to their water solubility; we mainly focused on the melanin which functions as the structural components of the *O. sinensis* in this study.

*O. sinensis* is naturally distributed on the high-altitude Qinghai–Tibet Plateau, where the UV level is very high because of the high altitude. Melanin pigments can protect fungi cells against UV radiation because of their ability to absorb a broad spectrum of electromagnetic waves [5]. In this study, the yeast experiments demonstrated that *O. sinensis* melanin has a strong UV-blocking ability, which gives it the potential to be applied in sunscreens. In addition to causing DNA damage, UV radiation can also cause harm to organisms by inducing carcinogenic ROS. The water-insoluble melanin derived from *I. hispidus* can clear intracellular ROS to enhance the cell viability of LO2 liver cells under H_2_O_2_ treatment, and shows strong antioxidant activity [9]. *O. sinensis* melanin also exhibits similar activity, which can neutralize ROS in various cells and inhibit cell apoptosis induced by H_2_O_2_ in HEK293 cells. It is generally accepted that the antioxidant activity of *O. sinensis* is attributed to peptides and polysaccharides. In this study, the cell experiments showed that *O. sinensis* melanin also has a strong ability to counteract intracellular ROS and its harmful activities, indicating that *O. sinensis* melanin is also one of the active components of *O. sinensis* that cannot be ignored.

## 4. Materials and Methods

### 4.1. Strain Cultivation and Melanin Extraction

*O. sinensis* was isolated from the wild Chinese cordyceps (Kangding, Sichuan, China), and preserved at the State Key Laboratory of Resource Insects (Southwest University, Beibei, Chongqing, China). *O. sinensis* was activated and cultivated in PDB medium (20% potato infusion and 2% glucose), shaken at 16 °C and 100 rpm for 40 days. The blastospore suspension was collected as seed solution with lens wiping paper and inoculated in OS1 liquid medium (20% potato infusion, 2% glucose, 1% yeast extract, 0.5% peptone, 0.1% KH_2_PO_4_, and 0.025% MgSO_4_), and cultured at 16 °C and 120 rpm. Light stress, ROS stress (induced using 100 μmol/L H_2_O_2_), and RNS stress (induced using 100 μmol/L sodium nitroprusside, SNP) were applied to observe the formation of melanin.

After fermentation, NaOH was used to bring the pH to 14 and mixed overnight at 100 °C using a magnetic stirrer. Afterward, the supernatant solution was collected using centrifugation at 10,000× *g* for 30 min, and then HCl solution (37%) was used to adjust the pH to 2 for 12 h. The crude melanin was collected using centrifugation at 10,000× *g* for 30 min and washed with ddH_2_O to pH = 7. This step was repeated three times to remove the proteins, carbohydrates, and lipids associated with melanin. Similarly, dichloromethane, ethyl acetate, methanol, ethanol, and water were used to remove impurities. Pure melanin was obtained using freeze-drying.

### 4.2. Elemental Analysis

The percentage contents of C, H, N, S, and O of 2 mg melanin were determined using an elemental analyzer (Thermo Scientific FlashSmart^TM^, Waltham, MA, USA).

### 4.3. Spectroscopy Analysis

#### 4.3.1. UV-Visible Absorption Spectrum

The *O. sinensis* melanin was dissolved in 0.1 M NaOH and the UV-visible absorption spectrum was scanned in the wavelength range of 190–600 nm by SpectraMax^®^ plus384 (Molecular Devices, Shanghai, China).

#### 4.3.2. Fourier Transform Infrared (FTIR) 

Melanin extracted from *O. sinensis* and qualitative standard (M832392-25mg, Macklin, Shanghai, China) was mixed with KBr (1:200) and pressed into tablets. Then, samples were scanned with FTIR (PE Spectrum Two, Waltham, MA, USA) in the scanning range of 4000–400 cm^−1^.

#### 4.3.3. Electron Paramagnetic Resonance (EPR)

The *O. sinensis* melanin was placed in a quartz tube and the EPR analysis was recorded using EMX Nano10602 (Bruker, Germany) at a temperature of 25 °C and a frequency of 9.645 GHz.

### 4.4. Pyrolysis Gas Chromatography and Mass Spectrometry (Py-GCMS)

The *O. sinensis* melanin was lysed using the Frontier EGA/PY3030D thermal cracking apparatus (Frontier, Fukushima, Japan), and the products were analyzed using GCMS-QP2020 (SHIMADZU, Kyoto, Japan). The NIST database was used for qualitative searching.

The analysis conditions were as follows: the pyrolyzer was set at 550 °C. The GC/MS analytical column was TG-5silMS 30 m × 0.25 mm × 0.25 µm. The GC oven temperature was operated from 60 °C (isothermal for 2 min) to 320 °C at a rate of 20 °C/min, and then kept isothermal for 13 min. The GC injector was maintained at 230 °C. The ionization method was electron ionization, and the ionization source temperature was 230 °C. The acquisition method was “scan” and the mass range was *m*/*z* 29–600 amu.

### 4.5. Biological Activity

#### 4.5.1. Ability to Chelate Metal Ions

Metal salt solutions (S1) of 20 mM CrCl_3_‧6H_2_O, MnCl_2_‧4H_2_O, FeSO_4_‧7H_2_O, CuSO_4_, ZnCl_2_, CdCl_2_‧5/2H_2_O, and Pb(Ac)_2_‧3H_2_O were each combined with 1 mg of melanin powder and incubated at 24°C overnight. Next, each mixture was centrifuged at 10,000× *g* for 10 min, the supernatant (S2) and precipitate (M) were collected, and the precipitate was dissolved in dimethyl sulfoxide (DMSO). Solution S1 or S2 was mixed in a YPD agar plate at a volume ratio of 1:15, and *Saccharomyces cerevisiae* was inoculated to observe the stress effect of the metal ions.

The metal ion concentrations in the preincubation (S1) and postincubation (S2) metal salt solutions and the postincubation melanin precipitation (DMSO dissolution) were determined using an atomic absorption spectrophotometer (ZA3300 FLAME AAS, Hitachi, Japan).

#### 4.5.2. UV-Blocking

*S. cerevisiae* was cultured in a YPD medium to an OD_600_ = 1.0, and the cells were harvested via centrifugation at 700× *g* for 5 min, washed twice with sterile water, and resuspended in sterile water to an OD_600_ = 1.5. The yeast suspension was placed in a sterile culture dish, melanin (dissolved in DMSO) was added to the final concentration of 0.1 mg/mL, and an equal volume of DMSO was added as a control. The two groups of yeast were placed under UV light (F8T5GL-Z287 15w, Forbens, Guangzhou, China), irradiated for different times, subjected to 10 times gradient dilution, and then dripped onto a YPD plate medium. The plates were placed at 28 °C.

#### 4.5.3. Free-Radical-Scavenging Ability

The ability of *O. sinensis* melanin to scavenge free radicals was measured using the DPPH method, and the procedure was adapted from Surendirakumar et al. [23]. Briefly, different concentrations of *O. sinensis* melanin were prepared with DMSO, and 0.05 mg/mL DPPH (ethanol solution) was mixed at a volume ratio of 1:1. After incubation in the dark at 24 °C for 30 min, a spectrophotometer (SpectraMax i3x, Molecular Devices, Shanghai, China) was used to read the absorbance at 517 nm. Trolox was used as a control. The antioxidant activity of *O. sinensis* melanin was also examined using the ABTS method as described by Khemakhem et al. [41]. Approximately 7.4 mM ABTS storage solution and 2.6 mM K_2_S_2_O_8_ storage solution were prepared, mixed with equal volumes, and reacted at 4 °C for 12–15 h (protected from light), and then diluted with ethanol to OD734 = 0.7 to obtain the ABTS working solution. The samples and ABTS working solution were mixed at a volume ratio of 1:4, kept in the dark at 24 °C for 30 min to react, and a spectrophotometer (SpectraMax i3x, Molecular Devices, Shanghai, China) was used to read the absorbance at 714 nm. 

The ability to scavenge the DPPH (or ABTS) radical was calculated using the following equation:

Scavenging rate (%) = [1 − (A_1_ − A_2_)/A_0_] × 100

where A_0_: DPPH (or ABTS)+ DMSO; A_1_: DPPH (or ABTS)+ melanin; and A_2_: melanin + ethanol.

#### 4.5.4. Antioxidant Activity on Cells

Human hepatocellular carcinoma (HepG2) cell line (HB-8065, ATCC), mouse leukemia cells of monocyte-macrophage (RAW264.7) cell line (TIB-71, ATCC), dendritic (DC2.4) cell line (HTX2245, ATCC), human colon cancer (SW620) cell line (CCL-227, ATCC), and human embryonic kidney 293 (HEK293) cell line (CRL-1573, ATCC) were purchased from the American Type Culture Collection and grown in complete growth medium supplemented with 10% fetal bovine serum (Gibco, Thermo Fisher Scientific, Waltham, MA, USA) at 37 °C with 5% CO_2_.

The intracellular ROS levels were measured using a ROS assay kit (Beyotime Biotechnology, Nantong, China). Briefly, the cells were inoculated into 24-well plates at 10^5^ cells per well, and melanin, DMSO (solvent control), and Trolox (positive control) were added. After 6 h, 50 μg/mL Rosup was used to induce ROS. Following the treatment, the cells were incubated with DCFH-DA for 20 min at 37 °C and then observed using fluorescence microscopy (ZESS Axio Observer A1, Oberkochen, Germany). The relative fluorescence intensity was then calculated and analyzed using GraphPad Prism (version 6.01) for the *t*-test.

The HEK293 cells were inoculated into six-well plates at 2 × 10^5^ cells per well, and melanin and DMSO (solvent control) were added. Following the method of Bian et al. [42], after 12 h, 0.5 mM and 1 mM H_2_O_2_ were used to induce oxidative damage to the cells. Western blotting was used to detect P53 (anti-P53, sc-126, Santa Cruz, Dallas, TX, USA), caspase-3 (anti-caspase-3, AF1213, Beyotime, China), Bax (anti-P53, sc-20067, Santa Cruz Dallas, TX, USA), and Bcl-2 (anti-Bcl-2, sc-7382, Santa Cruz Dallas, TX, USA) to characterize the apoptosis of the cells. Specifically, the cells were collected using centrifugation at 600× *g* for 5 min and lysed in RIPA buffer with a protease inhibitor cocktail (P1050, Beyotime, China). The cell lysates were centrifuged for 15 min at 12,000× *g* and 4 °C to sediment cell debris after 10 min incubation on ice. The total proteins were separated using sodium dodecyl sulfate-polyacrylamide gel electrophoresis, transferred to a polyvinylidene difluoride membrane, and blocked with 5% skimmed milk for 1 h. The membranes were then probed with primary antibodies overnight at 4 °C, followed by the secondary antibody for 2 h at room temperature. The electrochemical luminescence reagent (Thermo Fisher Scientific, USA) was used for immune detection and visualization using the Azure Biosystems C300 imaging system (Azure Biosystems, Dublin, CA, USA). GAPDH (anti-GAPDH, AF1186, Beyotime) was used as a control. The developed Western blot bands were measured using ImageJ (1.52V), and the values were analyzed using GraphPad Prism (version 6.01) for the *t*-test.

### 4.6. Statistical Analysis

The experimental data were analyzed using OriginPro (2022b SR1 9.9.5.171) statistical software. The measured data (x ± SD) are presented as the mean ± standard deviation. The significance of the data was analyzed using a one-way analysis of variance; *p* < 0.05 represents a significant difference.

## 5. Conclusions

In this study, *O. sinensis* melanin was obtained through liquid fermentation, and its structure and biological activity were studied. The yield of melanin could be increased by applying light or oxidative stress (ROS or RNS) during liquid fermentation. *O. sinensis* melanin has a maximum absorption value of 237 nm and contains a small amount of the sulfur element. The elemental and Py-GCMS analysis results demonstrated that it contains the benzene, indole, and pyrrole groups, which conforms to the typical characteristics of true melanin. It was found that *O. sinensis* melanin has strong UV-blocking activity and broad-spectrum heavy-metal-chelating activity. In addition, our study demonstrated that *O. sinensis* melanin has strong in vitro antioxidant activity, which may eliminate intracellular ROS and lessen oxidative damage brought on by H_2_O_2_. This study enriched the characterization of *O. sinensis* melanin structure and explored its biological activity, providing a theoretical basis for its application such as radiation resistance, heavy metal pollution remediation, and antioxidant use.

## Figures and Tables

**Table 1:** Comparison of elemental analysis of *O. sinensis* melanin and other fungi.

	C	H	O	N	S	C/N	C/H	C/O
*O. sinensis*	50.59	6.18	33.90	8.19	1.20	7.21	9.55	1.99
Eumelanin * [21]	56.45	3.15	31.82	8.49	0.09	7.76	20.91	2.37
Phaeomelanin * [21]	46.24	4.46	30.16	9.36	9.78	5.76	12.10	2.04
*Inonotus hispidus* [9]	43.67	6.25	46.58	3.5	0	14.56	8.15	1.25
*Ganoderma lucidum* [3]	54.20	6.24	33.35	5.14	1.07	12.30	10.13	2.17
*Phoma* sp. RDSE17 [22]	56.71	5.15	37.52	0.62	0	106.71	12.85	2.02
*Auricularia auricula* [23]	41.18	5.56	51.60	1.66	0	28.94	8.64	1.06
*Termitomyces albuminosus* [24]	54.68	3.54	26.92	2.49	12.36	25.60	18.00	2.71
*Boletus griseus* [25]	56.38	5.86	28.04	6.17	2.44	10.66	11.22	2.68

* The data for eumelanin and phaeomelanin are sourced from artificially synthesized dopa-melanin. Data on other fungal melanins are derived from the literature.

**Table 2:** The Py-GCMS products of *O. sinensis* melanin.

Num.	RT (min)	Area (%)	Structure	Num.	RT (min)	Area (%)	Structure
1	1.389	29.3	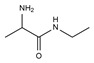	13	3.376	2.86	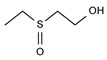
2	1.448	15.29	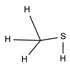	14	5.607	1.12	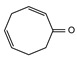
3	1.554	16.04	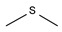	15	7.098	0.19	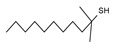
4	1.635	0.93	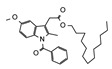	16	8.712	0.34	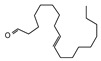
5	1.708	2.45	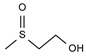	17	12.022	0.42	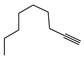
6	1.742	1.46	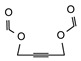	18	12.339	3.18	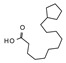
7	1.943	1.03	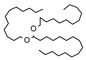	19	12.419	0.65	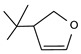
8	1.991	0.81	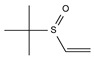	20	13.287	10.24	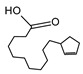
9	2.094	1.57	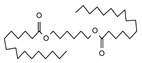	21	13.4	0.83	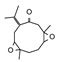
10	2.23	1.09	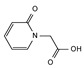	22	14.158	1.24	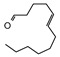
11	2.537	3.51	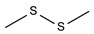	23	14.259	1.18	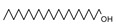
12	2.758	2.87	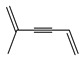	24	17.467	1.39	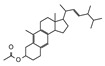

## Data Availability

All data are provided in the manuscript.
